# Privacy protected graphical functionality in DataSHIELD

**DOI:** 10.23889/ijpds.v1i1.296

**Published:** 2017-04-19

**Authors:** Demetris Avraam, Amadou Gaye, Julia Isaeva, Thomas Burton, Rebecca Wilson, Andrew Turner, Paul Burton

**Affiliations:** University of Bristol; Norwegian Institute of Public Health

## Objectives

In several disciplines such as in biomedicine and social sciences the analysis of individual-level data or the co-analysis of data from different studies requires the pooling and the sharing of those data. However, sharing and combining sensitive individual-level data is often prohibited by ethico-legal constraints and other barriers such as the control maintenance and the huge sample sizes. The graphical illustration of microdata is also often forbidden as can potentially be unsecured on the identification of sensitive information. For example the plot of a standard scatterplot is disclosive as can explicitly specify the exact values of two measurements for each single individual.

## Approach

DataSHIELD (www.datashield.ac.uk) is a novel approach that allows the analysis of sensitive individual-level data and the co-analysis of such data from several studies simultaneously without physically pooling the data.

## Results

DataSHIELD functionality consists of several functions that provide the flexibility of performing data analysis through different statistical techniques. A part of this environment includes a number of graphical-related functions for the graphical illustration of the statistical properties and relationships between different variables. We overview the graphical functions in DataSHIELD (ds.histogram, ds.heatmapPlot, ds.contourPlot) and demonstrate a number of new functions including ds.scatterPlot and ds.boxPlot developed based on the application of different computational approaches like the k-Nearest Neighbours algorithm and ensuring privacy protected analysis.

## Conclusion

DataSHIELD graphical functionality has certain methodological features for the representation of the relationships between different variables preserving their statistical properties and assuring the data privacy protection. These graphical approaches can be used or enhanced for application in various areas where confidentiality and information sensitivity is considered, for example in longitudinal data and survival analysis, in epidemiological studies, in geospatial analysis and several others.

